# Characteristics of Memory B Cells Elicited by a Highly Efficacious HPV Vaccine in Subjects with No Pre-existing Immunity

**DOI:** 10.1371/journal.ppat.1004461

**Published:** 2014-10-16

**Authors:** Erin M. Scherer, Robin A. Smith, Cassandra A. Simonich, Nixon Niyonzima, Joseph J. Carter, Denise A. Galloway

**Affiliations:** 1 Human Biology Division, Fred Hutchinson Cancer Research Center, Seattle, Washington, United States of America; 2 Department of Medicine, University of Washington, Seattle, Washington, United States of America; 3 Molecular and Cellular Biology Graduate Program, University of Washington, Seattle, Washington, United States of America; 4 Uganda Cancer Institute, Kampala, Uganda; 5 Department of Microbiology, University of Washington, Seattle, Washington, United States of America; National Cancer Institute, United States of America

## Abstract

Licensed human papillomavirus (HPV) vaccines provide near complete protection against the types of HPV that most commonly cause anogenital and oropharyngeal cancers (HPV 16 and 18) when administered to individuals naive to these types. These vaccines, like most other prophylactic vaccines, appear to protect by generating antibodies. However, almost nothing is known about the immunological memory that forms following HPV vaccination, which is required for long-term immunity. Here, we have identified and isolated HPV 16-specific memory B cells from female adolescents and young women who received the quadrivalent HPV vaccine in the absence of pre-existing immunity, using fluorescently conjugated HPV 16 pseudoviruses to label antigen receptors on the surface of memory B cells. Antibodies cloned and expressed from these singly sorted HPV 16-pseudovirus labeled memory B cells were predominantly IgG (>IgA>IgM), utilized diverse variable genes, and potently neutralized HPV 16 pseudoviruses *in vitro* despite possessing only average levels of somatic mutation. These findings suggest that the quadrivalent HPV vaccine provides an excellent model for studying the development of B cell memory; and, in the context of what is known about memory B cells elicited by influenza vaccination/infection, HIV-1 infection, or tetanus toxoid vaccination, indicates that extensive somatic hypermutation is not required to achieve potent vaccine-specific neutralizing antibody responses.

## Author Summary

There is an urgent need to better understand how to reliably generate effective vaccines, particularly subunit vaccines, as certain pathogens are considered to pose too great of a safety risk to be developed as live, attenuated or killed vaccines (e.g., HIV-1). The human papillomavirus (HPV) vaccines are two of the most effective subunit vaccines ever developed and have continued to show protection against HPV associated disease up to and beyond five years post-vaccination. Moreover, the target population for these vaccines have essentially no pre-existing immunity to the HPV types covered by the vaccine; therefore, these vaccines provide an excellent model for studying the immunity elicited by a highly effective subunit vaccine. As the HPV vaccines, like most vaccines, protect by generating antibodies, we are interested in characterizing the memory B cells elicited by the HPV vaccine. Memory B cells help to sustain antibody levels over time by rapidly differentiating into antibody secreting cells upon pathogen re-exposure. Although previous studies have provided evidence that the HPV vaccines elicit memory B cells, they did not characterize these cells. Here, we have isolated HPV-specific memory B cells from adolescent females and women who received the quadrivalent HPV vaccine and have cloned antibodies from these cells. Importantly, we find that these antibodies potently inhibit HPV and that the memory B cells from which they derive exhibit hallmarks of long-lived memory B cells.

## Introduction

The quadrivalent HPV (qHPV) vaccine provides near-complete protection against sexually transmitted HPV infections that most commonly cause anogenital and oropharyngeal cancers (HPV types 16 and 18) and genital warts (HPV 6 and 11) when administered to individuals naive to these types [1]–[6]. It is thus recommended as an adolescent vaccine or before the onset of sexual activity. The vaccine is comprised of virus-like particles (VLPs) assembled from the major capsid L1 protein of each of these four HPV types in alum. Although no correlate of protection has been confirmed for the qHPV vaccine due to low numbers of disease cases in vaccinees [7], passively transferred immune sera have been shown to be sufficient for protection against papillomavirus challenge in a number of animal models [8]–[10]. These findings suggest that the qHPV vaccine, like most prophylactic vaccines, protects by generating antibody (Ab) [11]. There is also evidence that qHPV vaccination elicits plasma cells, which help sustain antigen (Ag)-specific Ab levels over time by secreting Ab for extremely long periods; and memory B cells (Bmem), which renew Ab levels by rapidly differentiating into short-lived Ab-secreting plasmablasts upon re-exposure to Ag [12]. Classic plasma cells and Bmem are the products of germinal centers, which are transient structures that develop within secondary lymphoid tissues during a T-cell dependent immune response. It is also within germinal centers that B cell immunoglobulin genes undergo class-switching and somatic hypermutation, and where the resulting B cell receptors, or membrane tethered immunoglobulins, are selected for increased antigen affinity.

Evidence that qHPV vaccination elicits both plasma cells and Bmem derives from studies that have observed sustained Ab levels out to 5-years post-vaccination and boosts in Ab responses upon re-vaccination or *ex vivo* Ag exposure [13]–[15]. However, these studies have never directly identified or characterized HPV-specific Bmem. Such information would not only enable us to evaluate whether there are differences in the quality of B cell memory between different vaccine formulations or schedules, but would also advance our basic understanding of the immunological memory elicited by a highly efficacious vaccine in the absence of pre-existing immunity. The latter is particularly valuable to vaccine development, given that the target populations for candidate HIV-1 or hepatitis C virus vaccines have no pre-existing immunity to these infections.

Therefore, we developed an Ag-labeling method that uses fluorescently conjugated HPV 16 pseudoviruses (psV) to identify and isolate HPV 16-specific Bmem from the blood of female adolescents and young women who had no pre-existing HPV 16 immunity when they received the qHPV vaccine. Similar Ag-specific labeling approaches have been successfully employed by many groups to identify Bmem *ex vivo* [16]–[18]. We then cloned Abs from singly sorted HPV 16-psV labeled Bmem in order to evaluate whether these Bmem encoded functional Abs and/or exhibited hallmarks of classical Bmem (e.g., somatic hypermutation and class-switching). We chose to focus our studies on HPV 16, as it causes the vast majority of HPV-associated cancers [4].

## Results

### Generating and characterizing AF488-conjugated HPV 16 psV

In order to identify qHPV-specific Bmem, we generated Alexa Fluor 488 (AF488)-conjugated HPV 16 psV to fluorescently label Ag receptors on the surface of Bmem. These psV are comprised of the L1 and L2 (major and minor) capsid proteins of HPV 16 and a reporter plasmid, where L1 is the qHPV vaccine Ag. We chose psV as our ‘bait’ Ag not only because they contain the vaccine Ag in a polyvalent geometry that closely resembles the vaccine VLPs [19], but also because they could be evaluated for proper folding and function following AF488-conjugation, using a reporter-based neutralization assay [20]. In this assay, psV bound and neutralized by type-specific monoclonal Abs (mAbs) are prevented from entering 293TT cells and expressing an encapsidated reporter plasmid. Ab neutralization thus results in a decrease in reporter signal, which can be quantified. Both AF488-conjugated HPV 16 psV (AF488-HPV 16) and unconjugated HPV 16 psV are equally neutralized by an anti-HPV 16 murine mAb (**Fig. S1A**). This result indicates that AF488-HPV 16 are in the appropriate conformation, and that covalently linked AF488 moieties do not prevent Ab recognition. We observed the same result for AF488-conjugated and unconjugated bovine papillomavirus (BPV) psV, which were generated as negative controls for non-specific binding in our flow cytometry and neutralization assays, respectively. In addition, we found that AF488-HPV 16 and AF488-BPV psV exhibit essentially identical fluorescence intensities when applied to 293TT cells in optimized amounts (**Fig. S1B**).

### AF488-HPV 16 psV selectively identified Bmem post-vaccination

After confirming our AF488-psV reagents, we tested their ability to identify HPV 16-specific Bmem in peripheral blood mononuclear cell (PBMC) samples collected at month 7 (one month post-vaccination) from 12 qHPV vaccinees. As Ag-specific Bmem are rare [21], each sample was first enriched for B cells. These B cells were then further divided into two parts and separately stained with a multicolor flow cytometry panel to detect classic CD27^+^IgD^−^ Bmem [22], [23] and either AF488-HPV 16 or AF488-BPV. The frequency of AF488^+^ Bmem in each sample part was then analyzed by flow cytometry, and AF488-HPV16^+^ Bmem were single cell sorted into 96-well PCR plates.

**Figure 1:**
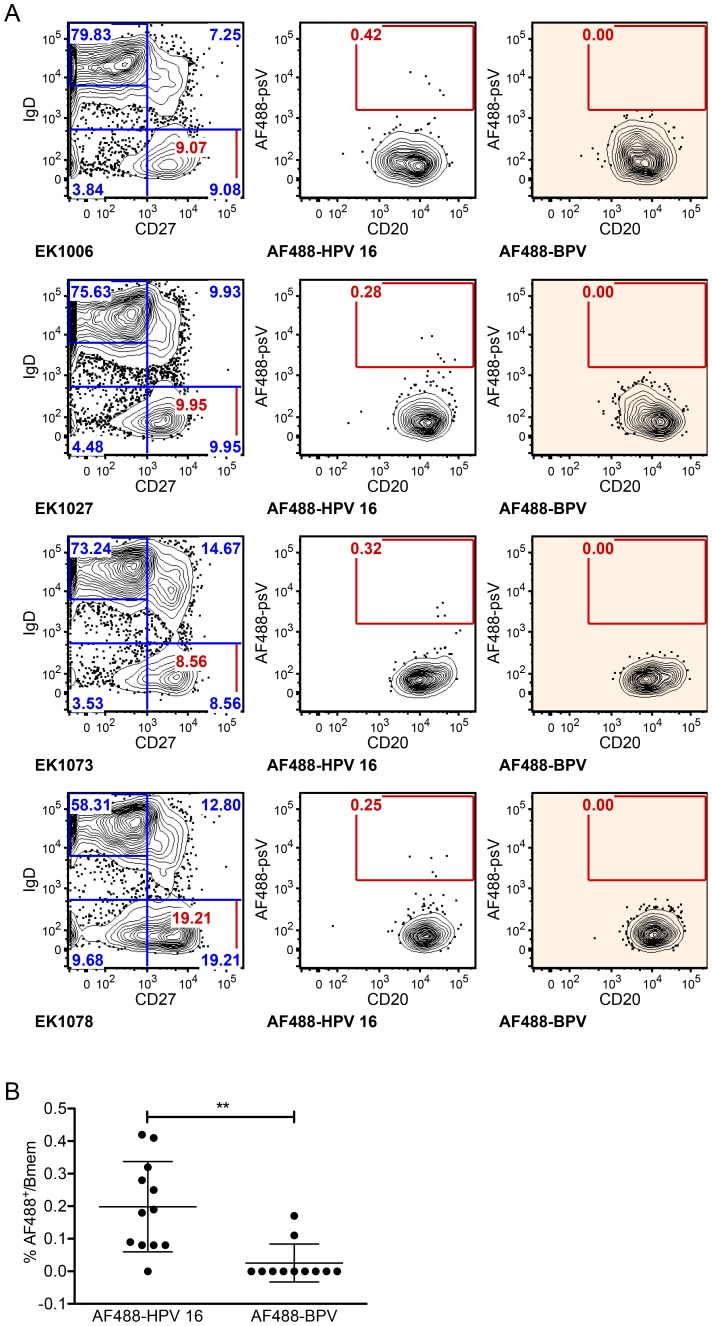
HPV 16-specific Bmem represent 0.2% of total Bmem in qHPV vaccinees at month 7. PBMC samples collected at month 7 (n = 12) were enriched for B cells, divided into two parts, and stained with a multicolor flow cytometry panel to identify classic Bmem and either AF488-BPV or AF488-HPV 16. (**A**) Representative dot plots are shown of the total naive B cell (IgD^+^CD27^−^) and Bmem (IgD^−^CD27^+^) frequencies in samples collected from subjects EK1006, EK1027, EK1073 and EK1078 (left column), as well as the frequencies of AF488^+^ Bmem observed by Ag-specific labeling (middle column) or by negative control labeling (right column). Flow cytometry data were first gated for cell size (forward versus side scatter), to exclude doublets and dead cells, and to include B cells (CD3^−^CD19^+^) (not shown). (**B**) AF488-HPV16^+^ Bmem were observed at a significantly higher frequency than AF488-BPV^+^ Bmem for all subjects' analyzed (**, p<0.005; paired, two-tailed student's t-test).

Representative flow cytometry data for 4 of 12 subjects post-vaccination show that AF488-HPV 16^+^ Bmem were clearly distinguishable from background, despite having very low cell numbers (only 1–10 cells sorted for each of nine samples; **Fig. 1A**). In contrast, almost no AF488-BPV^+^ Bmem were observed. Indeed, a significantly higher frequency of AF488-HPV 16^+^ Bmem than AF488-BPV^+^ Bmem were detected in all samples (**Fig. 1B**), thus confirming that AF488-HPV 16^+^ Bmem were not simply due to non-specific psV binding. Altogether, HPV 16-specific Bmem comprised ∼0.2% of total Bmem at one-month post-qHPV vaccination, and 29 AF488-HPV 16^+^ Bmem were singly sorted from nine different subjects' samples.

### Sequence characteristics of Abs cloned from HPV 16-specific Bmem

To evaluate the clonal diversity of HPV 16-specific Bmem elicited by the qHPV vaccine, we cloned full-length variable region sequences from the 29 singly sorted AF488-HPV 16^+^ Bmem described above. We designed primers that provide complete coverage of all known functional human leader and constant region 5′ exons that flank the variable region, as these regions exhibit low somatic hypermutation rates. The resulting materials and methods clone both the leader and variable regions from the heavy and light chains of single B cells as described in the **Methods**, **Figure S2**, and **Table S1**. For comparison, the *in silico* coverage of other commonly used primer sets that target the leader region are shown in **Figures S3, S4, S5**.

**Figure 2:**
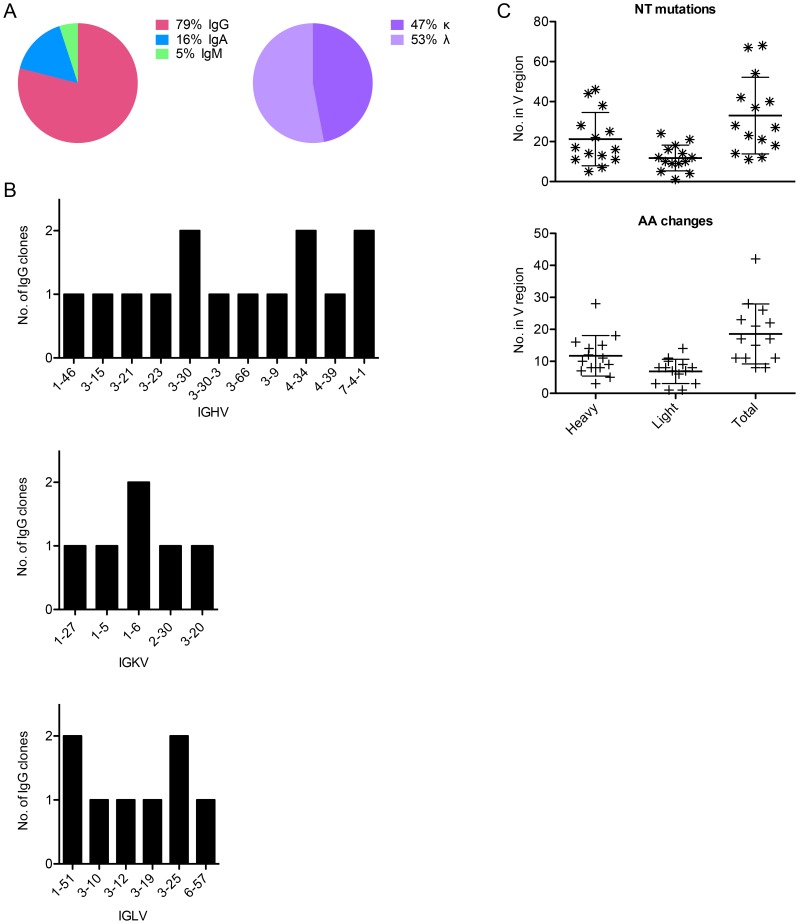
Sequence characteristics of Abs cloned from HPV 16-specific Bmem. (**A**) The isotype distribution of heavy chain sequences cloned from 29 singly sorted AF488-HPV 16^+^ Bmem is shown. There was approximately equal usage of kappa and lambda chains among IgG^+^ Bmem. (**B**) The number of unique IgG cloned with a given heavy or light chain V gene as determined by V-QUEST and IgBlast sequence analyses is plotted on the y-axis, where IGHV is immunoglobulin (Ig) heavy chain V gene; IGKV is Ig kappa chain V gene; and IGLV is Ig lambda chain V gene. (**C**) The number of nucleotide (NT) mutations and amino acid (AA) changes introduced into the heavy, light, and total V genes of each IgG is shown.

In total, 3 IgA, 15 IgG, and 1 IgM leader-variable regions were amplified from five different subjects' Bmem and confirmed through traditional DNA sequencing, of which approximately half were kappa and half lambda (**Fig. 2A**). This represents a PCR efficiency of ∼66% (19 Abs from 29 Bmem). The IgG antibodies were evaluated further, because they could be assessed for expression, folding, and function *in vitro*. Of these 15 clones, 14 generated high quality sequence reads that could be accurately analyzed for germline V, J, and D gene usage and somatic mutations using IMGT's V-QUEST [24] and IgBlast (**Table** **1**). We found that these 14 Abs derived from diverse V genes (**Fig. 2B**) and were somatically mutated (**Fig. 2C**), with an average of 33±19 NT mutations and 19±9 AA changes per clone. In cases where more than one IgG clone utilized a given V gene, it should be noted that the clones were derived from different subjects. Moreover, no clonally related IgG (or IgA, IgM) were observed in any subject, potentially due to low cell numbers.

**Table 1:** Sequence characteristics of Abs cloned from HPV 16-specific Bmem.

Ab	Subject	V gene^a^	J gene^a^	D gene^a^^,^ b	NT^a^^,^ c mutations	AA^a^^,^ d changes
1	EK1006	IGHV3-21*02	IGHJ4*02 or IGHJ5*02	IGHD6-13*01 or IGHD3-22*01	25	14
1	EK1006	IGKV1-6*01	IGKJ2*01		12	8
2	EK1027	IGHV3-66*02	IGHJ4*02	IGHD1-1*01 or IGHD1-1*01 and IGHD3-10*01	28	15
2	EK1027	IGKV2-30*01	IGKJ1*01		14	11
3	EK1027	IGHV1-46*01	IGHJ4*02	IGHD3-3*01	22	12
3	EK1027	IGKV1-6*01	IGKJ2*01		18	9
4^e^	EK1027	IGHV4-34*01	IGHJ6*03	IGHD6-19*01	11	7
4^e^	EK1027	IGLV3-12*01	IGLJ3*02		1	1
5	EK1042	IGHV3-9*01	IGHJ3*01	IGHD3-10*02	5	3
5	EK1042	IGLV3-19*01	IGLJ3*02		9	8
6	EK1377	IGHV3-30 *18	IGHJ4*02	IGHD4-17*01 or IGHD4-17*01 and IGDH3-3*02	7	5
6	EK1377	IGLV1-51*01	IGLJ1*01		4	3
7	EK1073	IGHV4-34*01	IGHJ6 *02	IGHD3-16*02 or IGHD3-16*02 and IGHD2-15*01	14	8
7	EK1073	IGLV1-51*01	IGLJ2*01 or IGLJ3*01		9	3
8	EK1073	IGHV3-15*01 or *04	IGHJ4*02	IGHD3-16*02	11	9
8	EK1073	IGKV3-20*01	IGKJ4*01		10	8
12	EK1073	IGHV3-30-3*01	IGHJ3*02	IGHD2-08*01 or IGHD2-8*01 and IGHD4-11*01	13	10
12	EK1073	IGKV1-5*03	IGKJ1*01		5	1
13	EK1073	IGHV3-23*04	IGHJ4*02	IGHD3-10*01 or IGHD2-21*01	38	16
13	EK1073	IGKV1-27*01	IGKJ1*01		16	7
16	EK1078	IGHV7-4-1*01 or *02	IGHJ4*02	IGHD5-12*01 or IGHD5-24*01	46	18
16	EK1078	IGLV3-25*03	IGLJ3*02		21	10
17	EK1078	IGHV7-4-1*02	IGHJ5*02	IGHD2-2*01 or IGHD2-2*01 and IGHD1-7*01	17	8
17	EK1078	IGLV6-57*01	IGLJ3*02		10	7
18	EK1078	IGHV3-30*18	IGHJ6*02	IGHD2-2*01 or IGHD5-5*01 or IGHD5-18*01	16	11
18	EK1078	IGLV3-25*03	IGLJ1*01		12	6
19^e^	EK1078	IGHV4-39*01	IGHJ4*02	IGHD3-9*01 or IGHD3-3*01 or IGHD2-8*01 or *02 or IGHD3-9*01 and IGHD1-1*01	44	28
19^e^	EK1078	IGLV3-10*01	IGLJ3*02		24	14

a determined using consensus results from IMGT/V-QUEST and IgBlast.

b The top D gene matches determined by both IMGT/V-QUEST and IgBlast are included.

If there were multiple matches, all of the top matches are shown for thoroughness.

c nucleotide.

d amino acid.

e potentially non-productive.

Heavy, kappa, and lambda chain sequences corresponding to 12 human monoclonal Abs (mAbs) were successfully cloned into their respective AbVec vectors using gene-specific forward and reverse cloning primers. Ab 1 was isolated from subject EK1006; Abs 2, 3, and 4 from EK1027; Ab 5 from EK1042; Ab 6 from EK1377; Abs 8, 12, and 13 from EK1073; and Abs 16, 17, and 18 from EK1078. We screened the resulting clones to identify those with 100% homology to the original PCR product, or, in rare cases where the original PCR product sequence was ambiguous, we used these clones to derive a consensus sequence. After co-transfecting corresponding heavy and light chain vectors into 293F cells to produce full-length IgG1, we found that IgG1 expressed with their native leader sequences gave comparable yields as an irrelevant IgG1 expressed with the murine leader encoded by the AbVec vectors (**Fig. S6**). In addition, we found that Ab 4 did not express, as predicted by V-QUEST.

### Abs cloned from HPV 16-specific Bmem exhibit potent neutralizing activities

**Figure 3:**
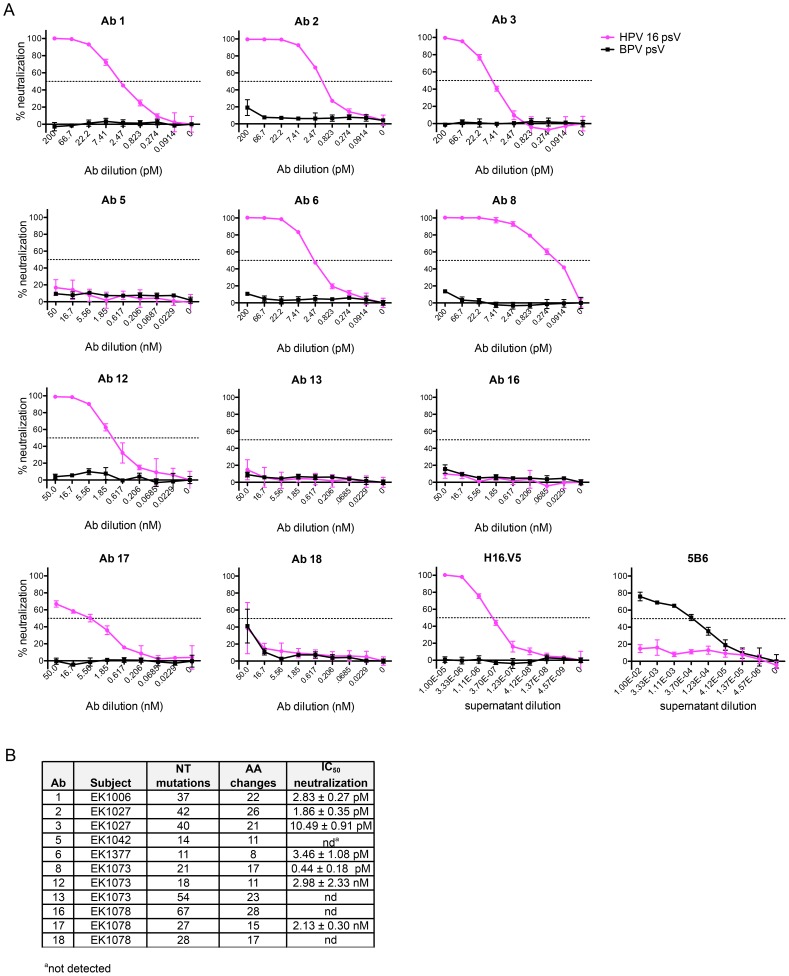
IgG cloned from HPV 16-specific Bmem are potently neutralizing. (**A**) Serial dilutions of human mAbs cloned from AF488-HPV 16^+^ Bmem (Ab 1, 2, 3, 5, 6, 8, 12, 13, 16, 17 and 18) were separately incubated in triplicate with a predetermined titer of unconjugated HPV 16 psV (solid pink lines) or BPV psV (negative control, solid black lines). All human mAbs were initially tested at a starting concentration of 50 nM (**Fig. S8**); however, if Abs neutralized HPV 16 at all dilution points (e.g., Ab 1–8), they were re-tested at a lower starting concentration to obtain precise IC_50_ values. The anti-HPV 16 murine mAb, H16.V5, and anti-BPV murine mAb, 5B6, served as positive controls for HPV 16 and BPV psV neutralization. HPV 16 and BPV psV are comprised of major and minor capsid proteins encompassing a secreted alkaline phosphatase (SEAP) reporter plasmid. After one hour at room temperature, the Ab-psV mixtures were transferred to 293TT cells and incubated for three additional days at 37°C. The amount of AP activity in the supernatant was then quantified, corrected for background, and expressed as percent neutralization, or the inverse of the AP signal reduction observed in the presence of Ab: [(mean_signal without Ab_−mean_signal with Ab_)/(mean_signal without Ab_)]×100. A dotted line indicates 50% neutralization; however, IC_50_ values shown in the adjacent table were derived from nonlinear regression analyses and represent the mean value (± SD) of two or more replicate experiments. (**B**) Table summarizing the characteristics of human mAbs cloned from HPV 16-specific Bmem.

To demonstrate that the low number of AF488-HPV 16^+^ Bmem found in the subjects' samples expressed functional Abs that were, in fact, HPV 16-specific, we evaluated our human mAbs in the aforementioned neutralization assay. We also included murine mAbs H16.V5 and 5B6 as well-characterized positive controls for HPV 16 and BPV neutralization, respectively [25]–[27]. A series of dilutions of each Ab were tested against both unconjugated HPV 16 psV (pink solid lines) and BPV psV (black solid lines) (**Fig. 3A**). From the neutralization curves it is immediately apparent that none of our human Abs neutralized BPV psV. In contrast, most of the human Abs neutralized HPV 16 psV with potent IC_50_ values ranging from 0.44 pM–2.98 nM (**Fig. 3B**). This result thus demonstrates the utility of our Ag-labeling approach for identifying HPV 16-specific Bmem.

**Figure 4:**
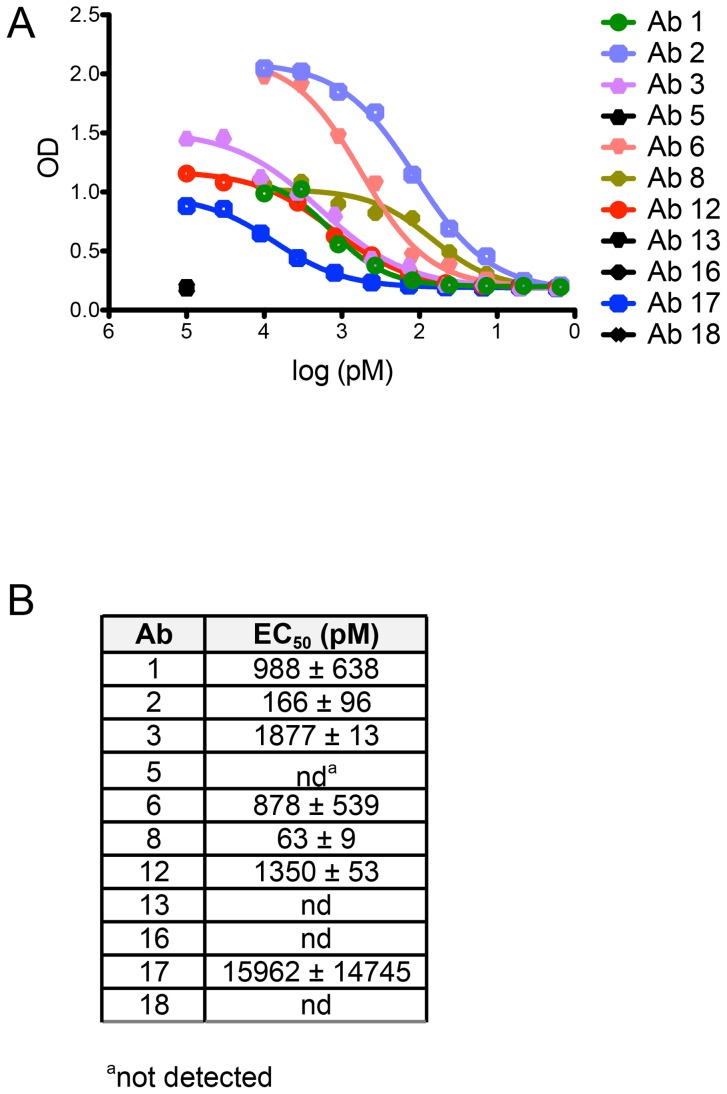
Non-neutralizing human mAbs 5, 13, 16, and 18 do not bind to HPV 16 psV. Each Ab cloned and expressed in this study was evaluated for binding to HPV 16 psV over a broad dilution range in an ELISA. (**A**) Representative binding curves from one experiment are plotted with optical density (OD) on the y-axis and Ab dilution on the x-axis. (**B**) Table summarizing the mean half-maximal binding concentrations, or EC_50_ values (± SD from two or more experiments), measured for Ab binding to HPV 16 psV. EC_50_ values were calculated using a non-linear regression model.

Four of the twelve human mAbs (5, 13, 16, and 18) did not neutralize HPV 16 even when tested at dilutions up to 450 nM. Therefore, to learn whether there are differences in the abilities of these Abs to bind vs. neutralize HPV 16, as has been observed for Abs elicited against other viruses (e.g., HIV-1), we evaluated their binding to psV in an ELISA. In this assay, antibodies in serum or plasma are tested for binding to psV immobilized on the surface of polystyrene microtiter plates. Antibody binding is then detected and quantified using a secondary antibody against human IgG. Similar to the neutralization results, we found that Abs 5, 13, 16, and 18 did not bind to HPV 16 L1 in the ELISA, even when tested at concentrations up to 100 nM (**Fig. 4A**). Therefore, it appears that these Abs are not HPV 16-specific and that the AF488-HPV 16+ Bmem from which they derived represented background or false positives. Such non-specific staining has been observed with other Ag-labeling methods [28].

We also found that the EC_50_ values for binding were higher than the IC_50_ values for neutralization, indicating that Ab affinities were lower in the ELISA than in the neutralization assay (**Fig. 4B**). Lower apparent Ab affinities in the ELISA than in the neutralization assay have also been observed for the anti-HPV 16 murine mAbs [25], [29]. This discrepancy was subsequently suggested to result from the ELISA conditions not satisfying the law of mass action, unlike the neutralization assay; therefore, the ELISA may be a less accurate measure of Ab affinity [25], [30].

## Discussion

In order to characterize Bmem elicited by the qHPV vaccine, we first needed to establish an Ag-labeling method to identify qHPV-specific Bmem. The AF488-psV-labeling method described here both successfully and selectively identifies Ag-experienced HPV 16-specific Bmem as evidenced by the findings that significantly more AF488-HPV 16^+^ than AF488-BPV^+^ Bmem were observed in the post-vaccination samples, and that Abs cloned from these cells potently neutralized HPV 16 psV. Such Ag-specificity is perhaps not surprising, given that HPV Ab responses are almost uniformly type-specific, and HPV 16 L1 only shares ∼77% amino acid identity with BPV L1 [31], [32]. Still, the use of a negative control (AF488-BPV) was likely instrumental in our high success rate for identifying HPV 16-specific Bmem. Taken all together, these results show that we can both find and characterize HPV 16-specific Bmem in qHPV vaccinees.

The observed frequency of HPV 16-specific Bmem is within the range of Ag-specific Bmem frequencies observed by other studies that have used Ag-labeling [33], [34]. However, it is nearly an order of magnitude lower than the frequency of HPV 16-specific Ab-secreting cells reported for these samples by Smolen *et al.* [35]. This discrepancy may be attributed to differences in both methodology and sampling. Specifically, Smolen *et al.* utilized an ELISPOT assay to quantify HPV 16-specific IgG secreting cells, and ELISPOT has been shown to be a more sensitive method for detecting Ag-specific responses than Ag-labeling [36]. It is also possible that we unintentionally excluded some HPV 16-specific Bmem by positively gating for singlet cells. In addition, the authors evaluated more samples than we did (166 vs. 12 samples); therefore, the frequency of HPV 16-specific Bmem reported here may actually underrepresent the total vaccine response. We recognize that the low numbers of HPV 16-specific Bmem observed in these samples may also have limited our findings. These low numbers derive in part from the low number of starting cells in each sample (7.8×10^6^±2.3×10^6^ PBMCs) and in part from diminished viability of these samples. In fact, based upon the observed average number of PBMCs, B cell frequency (∼5%), B cell purity (∼90%), sample viability (∼80%), Bmem frequency (∼10%), and frequency of HPV 16-specific Bmem (∼0.2%), we anticipated detecting at most 25 cells per sample.

We also needed to establish methods for cloning the Ag-specific receptors, or Abs, from these cells. We opted to clone Abs from single B cells in order to retain heavy and light chain pairing information. For the latter, we sought to identify primers that would amplify heavy and light chain variable regions with minimal bias by targeting region that exhibit low somatic hypermutation rates. However, in our search we discovered that many such published primer sets had gaps in coverage, which are particularly notable in the case of the lambda chain primers and have resulted in documented amplification biases [37], [38]. Therefore, we developed new heavy, kappa, and lambda chain primers to provide complete coverage of known human leader and constant region sequences. Although we have not directly compared all of these primer sets and their associated methods, we find that the efficiency of our primers and method for amplifying paired Ab sequences from Bmem (∼70%) is greater than that reported by other groups (∼20–50%), all of which are lower than the efficiencies reported for amplifying Ab sequences from plasmablasts (≥80%) [34], [39]. Such gross differences in amplification efficiencies likely reflect the disparate levels of Ab transcripts in these B cell subsets [40].

Isolated AF488-HPV 16^+^ Bmem were predominantly IgG (79%), but also included IgA (16%) and IgM (5%) Bmem. Although we did not observe any somatic variants in this study, Huo *et al.* have described the appearance of both HPV 16-specific IgA and IgG Ab-secreting cells (i.e., plasmablasts) in blood following qHPV vaccination [41]. Therefore, it will be interesting to see if we find clonally related HPV 16-specific IgA and IgG in our future work.

We also found that the IgG clones were somatically mutated (∼33 NT mutations and ∼19 AA changes from germline, on average) and utilized diverse germline V genes. This level of Ab somatic hypermutation is not remarkably high. Indeed, it is more similar to the level observed for IgG cloned from healthy donors, influenza vaccinees, or tetanus toxoid (TT) vaccinees than the broadly neutralizing anti-HIV-1 Abs described in the literature [34], [37], [42]–[45]. The former studies found an average of 18.0 and <10 NT mutations in the heavy and light chains of healthy donor IgG^+^ B cells; 25.3 and 16.2 NT mutations in the heavy and light chains of TT-specific Bmem; and 19.4 NT mutations in the heavy chain of plasmablasts responding to seasonal influenza vaccination. Moreover, Klein *et al.* have shown that anti-HIV-1 Abs with limited neutralization breadth have ∼40 NT mutations and ∼24 AA changes on average. In contrast, the most potent and broadly neutralizing anti-HIV-1 Abs have ∼118 NT mutations and ∼56 AA changes [46]. Our data thus appear to corroborate an emerging trend that extensive somatic hypermutation is not required to achieve potent, albeit narrow or type-specific, neutralizing Abs responses. Such differences may also reflect differences in antigen immunogenicity and/or the cumulative Ag- and germinal center-experience of the Bmem. Indeed, we know that broadly neutralizing Ab responses develop later in HIV-1-infected individuals than isolate-specific responses [47].

The correlation between somatic hypermutation levels and neutralization breadth may also indirectly relate to diverse V gene usage as opposed to the convergent V usage reported for both broadly neutralizing HIV-1 and influenza Abs [37], [44], [48]–[50]. Namely, there are many solutions (germline V genes) to a simple problem (neutralizing one virus); but fewer solutions to a more complex one (e.g., V genes that can tolerate framework mutations, which appear to be key for neutralizing many different viruses; [46]). It will thus be interesting to see if there are any differences between the extent of somatic hypermutation or V gene usage in Bmem repertories elicited by vaccination versus that observed in Bmem repertories generated following natural HPV infection.

Finally, it should be noted that we do not know whether the Abs expressed by these HPV 16-specific Bmem are present in serum. This may be important if pre-existing Bmem are not able to detect HPV and generate a *de novo* Ab response in time to neutralize the incoming virus. The kinetics of HPV infection occurs in hours [51], [52], whereas Bmem to plasmablast differentiation takes 4–8 days post-vaccination when measured in peripheral blood [39], [45], [53]. Therefore, it would appear that the concentration and affinity (i.e., composition) of pre-existing Abs may be more likely determinants of protection against HPV infection. At the same time, Bmem to plasmablast differentiation appears to occur more rapidly for certain vaccines (e.g., antibodies to the hepatitis B virus (HBV) surface Ag have been detected in sera at 3–4 days post-HBV vaccine boosting [54]) and may occur more rapidly at the site of infection or in draining lymph node(s) than can be measured systemically.

A recently published study may provide some indication of the overlap between Bmem and serolological Ab repertoires: Lavinder *et al.* showed that in the case of the tetanus toxoid (TT) vaccine, only a minor fraction of the Bmem and plasmablast Ab repertoires at day 7 post-boosting (<1% and <5% respectively) comprise the Ag-specific serological Ab repertoire found using proteomics [55]. This study thus indicates that not all plasmablast and/or Bmem clones are selected (or survive) to populate the bone marrow niche. Two potential confounders of this interpretation, however, were that Bmem analyzed in this study were not selected for Ag-specificity and were not sampled at the peak of the Bmem response, which occurs on day 14 post-TT boosting [39]. Frölich *et al.* assessed the kinetics and repertories of both TT-specific Bmem and TT-specific plasmablasts and found that the composition of their Ab repertoires were highly similar. Taken together, these two studies suggest that a low frequency of the Ag-specific Bmem repertoire (<5%) makes it into the serological Ab repertoire. Similarly, Purtha *et al.* showed that the Ab specificities of Bmem following west nile virus infection in mice were broader than the Ab specificities expressed by plasma cells or found in serum. However, these authors did not show the frequency of Bmem clones among the plasma cell and/or serological repertoires. Similar studies comparing Bmem and serological repertoires are warranted for other vaccines and infections. For although the TT vaccine is highly efficacious, its Ab levels decline steadily over time, suggesting that it does not elicit the same quality of long-lived plasma cells as the smallpox or yellow fever vaccines, for example [56].

## Materials and Methods

### Samples

De-identified PBMC samples were collected as part of a phase III, post-licensure, randomized, controlled, multi-center trial (NIH registry number NCT00501137) to compare the immunogenicity of a reduced dose qHPV vaccine schedule to that of the licensed 3-dose qHPV vaccine [57]. This trial included three study groups: female adolescents (aged 9–13 years) who received a 2-dose qHPV vaccine at months 0 and 6; female adolescents who received the 3-dose qHPV vaccine at months 0, 2, and 6; and young women (aged 16–26 years) who received the 3-dose qHPV vaccine. PBMC samples were isolated from pre-vaccination and post-vaccination blood draws as described [35].

### Generation of HPV 16 and BPV psV

HPV 16 and BPV psV were generated using plasmids and protocols described on the National Cancer Institute (NCI)'s Laboratory of Cellular Oncology (LCO) website (http://home.ccr.cancer.gov/lco/default.asp) with the following modifications: 45 million 293TT cells (NCI Developmental Therapeutics Program) were seeded overnight in each of three 225 cm^2^ tissue culture flasks (Corning; Sigma-Aldrich, St. Louis, Missouri) with 60 ml of DMEM-10 [high-glucose DMEM (Life Technologies, Carlsbad, California) supplemented with 10% FBS (Gemini Bioproducts, West Sacramento, California), L-glutamine, and 1× non-essential amino acids (Life Technologies)]. The next morning flasks between 70–90% confluency were each co-transfected with 57 µg of pYSEAP (plasmid expressing secreted alkaline phosphatase, or SEAP) and either 57 µg of p16L1L2 (plasmid expressing HPV 16 L1 and L2) or pBPVL1L2 (plasmid expressing BPV L1 and L2) using 255 µl of Lipofectamine2000 transfection reagent (Life Technologies). After ∼72 hours, cells were recovered by centrifugation and transferred to a siliconized eppendorf tube in phosphate buffered saline (PBS)-Mg buffer [PBS supplemented with 1× antibiotic-antimycotic (Life Technologies) and 9.5 mM MgCl_2_]. Cells were then re-suspended in PBS-Mg solution (1.5× the volume of cells), 0.5% Triton X-100 (Thermo Fisher Scientific, Rockford, Illinois), 40 mM sodium phosphate buffer pH 7.5, and 20 µg/ml RNAse A; and incubated for 24 hours at 37°C. Lysate was stored at −80°C until use.

### Conjugation and purification of Alexa Fluor 488 (AF488)-labeled psV

AF488 dye (Life Technologies) was re-suspended in 200 µl molecular biology grade (MB) water, aliquoted in eppendorf tubes, lyophilized, and stored at −20°C until use.

PsV were conjugated with AF488 using protocols described on the LCO website with the following specifications: Lysates prepared above were clarified by centrifugation (10 min at 5,000× g, 4°C), transferred into clean siliconized eppendorf tubes, re-clarified by centrifugation, and then pooled in polypropylene tubes. Lysate was diluted to 3.5 mg/ml protein in PBS and divided into 1 ml fractions in clean siliconized eppendorf tubes containing 100 µl of 1M sodium bicarbonate buffer, pH 8.5. Pre-aliquoted AF488 dye was thoroughly re-suspended in DMSO at 10 mg/ml and then 34 µg was added to each tube of diluted lysate and immediately vortexed. Samples were rotated end-over-end for one hour at room temperature while protected from light. Each tube of lysate was then brought to neutral pH with 40 µl of 1M sodium phosphate buffer, pH 6.5. Pooled tubes of neutral conjugated lysate were purified by density gradient ultracentrifugation using layered gradients of 27%, 33%, and 39% Optiprep (Sigma-Aldrich) and PBS as a diluent, as previously described [20].

Fractions collected following gradient ultracentrifugation were screened in duplicate for the presence of psV using a direct ELISA with H16.V5 and 5B6 (**Methods S1**). Fractions containing the highest signals above background were pooled, aliquoted, and stored at −80°C until use.

To determine the relative amounts of L1, and thus psV, in each AF488-HPV 16 and AF488-BPV stock, sample aliquots were reduced with 6.8% (v/v) 2-mercaptoethanol (Sigma-Aldrich) for 5 minutes at 100°C and separated on SDS-PAGE gels. Gels were then stained with Coomassie blue and L1 band (∼55 kDa) intensities quantified using ImageJ [58]. The average ratio of AF488-HPV 16:AF488-BPV L1 band intensities from duplicate gels was used to normalize AF488-psV amounts for subsequent flow cytometry experiments. AF488-HPV 16 and AF488-BPV were separately titrated on 293TT cells to identify optimal amounts of fluorescent Ag for B cell staining.

### B cell enrichment and staining

PBMCs were quickly thawed in pre-warmed, heat inactivated FBS, re-suspended in ice-cold MACS separation buffer (Milteyni Biotec, Auburn, California), counted, and enriched for B cells using the B Cell Isolation Kit II (Milteyni Biotec). Enriched B cells were then washed and re-suspended in PBS, divided in half, and stained with Live/Dead Violet dead cell dye (Life Technologies) for 30 minutes [59]. Each of the two samples were then washed and re-suspended in 2% FBS-PBS solution and stained with either AF488-HPV 16 psV or AF488-BPV psV, as well as the following fluorescent mAbs for 30 minutes: anti-CD38 APC and anti-IgD PE (Milteyni Biotec), anti-CD3 V500, anti-CD19 APC-Cy7, anti-CD27 PE-Cy7, and anti-CD20 PerCP-Cy5.5 (BD Biosciences, San Jose, California). All staining was conducted with optimized amounts of staining reagents, cells on ice, and minimal light exposure. Stained cells were washed and re-suspended in 2% FBS-PBS, maintained on ice, and protected from light until fluorescence activated cell sorting (FACS).

### Single cell sorting

FACS was conducted using an Aria II (BD Biosciences) in single cell sort mode. Just prior to FACS, sort buffer was prepared using a modified formulation of the buffer described by Wardemann *et al.*: 0.425× RNase-free PBS (Life Technologies), 10 mM dithiothreitol (dTT), and 16 U RNasin (Promega, Madison, Wisconsin) [60]. Eight µl of this buffer were added to each well of a 96-well AB-2800 PCR plate (Thermo Fisher Scientific), sealed with adhesive PCR films (Thermo Fisher Scientific), and kept on ice until sorting.

Cells were gated for size (SSC-A vs. FSC-A), to exclude doublets (SSC-W vs. SSC-H; FSC-W vs. FSC-H) and dead cells (Live/Dead Violet^−^), and to include Bmem (CD3^−^CD19^+^CD20^+^CD27^+^IgD^−^) with a high AF488-HPV 16^+^ fluorescence intensity above background (AF488^−^ cells in samples stained with AF488-BPV). For each well containing a single sorted cell, an equal number of wells were kept empty as non-template controls. Immediately after sorting, plates were sealed with foil PCR films (Thermo Fisher Scientific), placed onto dry ice, and stored at −80°C.

### Single B cell RT-PCR with random primers

Sort plates were thawed on ice and briefly centrifuged before adding 11.7 µl per well of ice-cold RT-PCR master mix containing 5.8 µl nuclease free water, 4.8 µl 5× FS buffer (Life Technologies), 1 µl 25 mM dNTP mix (Roche Applied Science, Indianapolis, Indiana), and 0.1 µl random primers (Life Technologies, catalog no. 48190-011) per well. Plates were again briefly centrifuged, incubated at 65°C for 5 minutes, and returned to ice. Then 8.3 µl of ice-cold RT-PCR master mix (2) containing 4.8 µl nuclease-free water, 0.8 µl 5× FS buffer, 0.2 µl RNasin, 2 µl 0.1M dTT, and 0.5 µl SuperScript III RT (Life Technologies) were added per well, and cDNA was generated with the following PCR program: 1 cycle for 5 minutes at 25°C, 1 cycle for 60 minutes at 50°C, 1 cycle for 15 minutes at 70°C, and 4°C forever. cDNA was stored at 4°C (short-term) or −20°C (long-term).

### Ab cloning primer design

In order to amplify full-length variable regions from singly sorted AF488-HPV 16^+^ Bmem with minimal bias, we designed forward amplification primers that anneal within the leader region, which precedes the variable region, and reverse amplification primers that anneal within the constant region, which follows the variable region and possesses substantially less germline and somatic variation than the variable region, as its name implies. In the case of the forward primers, we specifically designed them to anneal within the first of two exons in the Ab leader region, as the 5′ boundary of somatic hypermutation lies within the leader intron [61]–[64].

Where possible, up to two degenerate bases were used to minimize the number of primers in each set. Primers were evenly distributed across primer sets on the basis of similar melting temperatures (within ∼3°C of variation, according to the default analysis settings of Integrated DNA Technologies' OligoAnalyzer 3.1, Coralville, Iowa) and minimal primer-primer interactions (delta G≤−10 kcal/mol, base pairs ≤4; OligoAnalyzer 3.1). The resulting primer sets and all other primers used in this study are provided in **Table S1**. For example, we also designed forward and reverse cloning primers based upon the above amplification primers, so that the amplification PCR products could be recombinantly expressed as full-length Abs using the AbVec vectors [65].

Importantly, our forward amplification primers possess complete homology to all known functional human leader sequences for the heavy, kappa, and lambda chains from IMGT (**Fig. S2**). Our reverse primers also possess complete homology to all known human IgA, IgG, IgM, kappa, and lambda constant region alleles.

### Amplifying leader-variable regions from single B cells

To amplify full-length variable regions from the bulk cDNA generated above, six forward primer sets based on the sequences of all known functional Ab leaders were used: three for the heavy chain, two for the lambda chain, and one for the kappa chain (details of set-up are also included in **Table S1**). Each forward primer set was added separately to a given master mix from the reverse primer and other PCR components. The final concentration of the forward primer set within the master mix was 0.5 µM. Some of the forward primer sets contain degenerate primers. In this case, each degenerate primer was treated as *n* primer parts, where *n* represents the number of degenerate bases. For example, if a primer contains only one degenerate position, and that position is a ‘D’ nucleotide (D = A, C, or G), such a degenerate primer contains three degenerate bases and would be treated as three primer parts. There were equal nmoles of each primer part in the final master mix. The reverse primer was added in 3-fold excess of this amount (i.e., 3-fold the nmoles of each primer part).

In addition to the variable volumes of the forward primer set and reverse primer, the other PCR components per PCR plate well included: 3 µl of cDNA, 4.375 µl HotStar Taq Plus buffer, 2.5 µl MgCl_2_, 0.5 µl 25 mM dNTP mix, 0.4375 µl HotStar Taq Plus DNA Polymerase (Qiagen, Valencia, California), and nuclease-free water to 40 µl. The PCR program used was: 1 cycle for 5 minutes at 95°C; 50 cycles at 94°C for 30 seconds, 54–59°C for 30 seconds (as indicated in **Table S1**), and 72°C for 55 seconds; 1 cycle at 72°C for 10 minutes, and 4°C forever.

The IgG heavy and kappa chain PCR reactions were conducted first, and the resulting products analyzed on DNA agarose gels for the presence of ∼500 or ∼630 bp fragments, respectively. If no PCR product was observed for a given well or wells with the kappa chain primer set, additional PCR reactions were carried out for these wells using the two lambda chain primer sets (∼470 bp band expected). PCR products in wells with clearly visible Ab bands were then purified from the other PCR components using a QIAquick PCR purification kit (Qiagen), resuspended in MB water, and submitted for traditional DNA sequencing with the described sequencing primers. After the variable region sequences of these products were confirmed and assigned to their respective germline genes using IgBlast and V-QUEST, single gene-specific forward and reverse cloning primers were employed to re-amplify these variable regions for molecular cloning, using the same PCR conditions described above and 1 pg of template. The resulting products were again purified from other PCR components and resuspended in MB water.

### Cloning leader-variable region amplicons into Ab expression vectors

Abvec-hIgG (FJ475055.1), Abvec-hIglambda (FJ517647.1), and Abvec-hIgKappa (FJ475056.1) vectors were kindly provided by Patrick Wilson's lab (University of Chicago). Similar to other Ab expression vectors, the AbVec vectors encode IgG1, Igκ1, and Igλ2 constant regions in-frame and downstream of cloning sites for exogenous variable regions, as well as a murine leader in-frame and upstream of the variable region cloning sites. In their current configurations, these cloning sites trim off the very 5′, and in some cases also the 3′, end of inserted variable regions. However, as we wished to preserve the entire variable region sequence, we utilized alternative cloning sites and introduced a new cloning site in AbVec-hIgKappa at bps 1417–18 using site-directed mutagenesis. These sites allow both the leader *and* variable region sequences to be cloned into these vectors, including the first 4, 33, and 14 amino acids of the corresponding IgG CH1, Cκ, or Cλ. The leader-specific forward primers and IgG/κ/λ specific reverse primers for the cloning PCR reactions thus contain restriction enzyme sites corresponding to these alternative 5′/3′ cloning sites, which for AbVec-hIgG1, AbVec-hIgKappa, and AbVec-hIgLambda correspond to EcoRI/ApaI, EcoRI/XhoI, and EcoRI/XhoI, respectively. It should be noted that only in one case does the inserted coding sequence of the constant region allele (IGLC1*02) upstream of the cloning site differ from that of the vector allele (IGLC2*02). However, we have confirmed that in this case, the altered AAs do not alter the expression, folding, or neutralization potency of the resulting hybrid Ab clone when compared to ‘corrected’ clone (i.e., a clone where the constant region coding sequence upstream of the cloning site was reverted to the vector coding sequence; **Fig. S7**).

Heavy, kappa, and lambda chain PCR products generated above were inserted into each respective AbVec vector using traditional cloning techniques, and the resulting colonies were screened for the correct insert size and sequence.

In-Fusion cloning technology (Clontech, Mountain View, California) was utilized for sequences containing internal restriction sites.

### Recombinant Ab expression

To generate full-length Abs from the above cloning products, 15 µg of each heavy and light chain vector were co-transfected into Freestyle 293-F cells according to the manufacturer's protocols (Life Technologies). Cell supernatants were collected by centrifugation 72 hours post-transfection. IgG1 were purified from sterile-filtered supernatants using 0.3 ml of protein G agarose (0.6 ml slurry) and 0.5–2.0 ml capacity disposable plastic columns according to manufacturer's recommendations (Thermo Fisher Scientific), except that PBS was used as a binding buffer; 0.1M citric acid, pH 3.0 was used as an elution buffer; and 1M Tris base, pH 9.0 was used as a neutralization buffer. Following dialysis in PBS, Abs were sterile-filtered and stored at 4°C.

### HPV neutralization assay

The 293TT psV neutralization assay was conducted using protocols described on the LCO website, with the following specifications: Unconjugated HPV 16 and BPV psV were generated and purified as described for AF488-psV above, omitting the dilution of clarified lysate and AF488 conjugation steps. Four to six hours before the addition of Ab and psV mixtures to cells, 30,000 293TT cells were seeded in the inner 60 wells of a 96-well flat bottom tissue culture plate in 100 µl of DMEM-10++ [DMEM-10 supplemented with 1× pen strep (Gemini Bioproducts) and 400 µg/ml hygromycin B (Mediatech, Manassas, Virginia)]. The outer 36 wells were filled with 200 µl DMEM-10+ (DMEM-10 supplemented with 1× pen strep), and plates were returned to 37°C. Approximately two hours before Ab and psV mixtures were added to cells, three-fold Ab dilution series were prepared in triplicate in DMEM-10++ in 96-well polypropylene plates on ice, including H16.V5 and 5B6. Initially, the starting concentration of purified human mAbs was 167 nM (25 µg/ml) per well, but after subsequent experiments showed that these Abs were extremely potent, starting human mAb concentrations were reduced to 50 nM. PsV were also diluted in DMEM-10++ to a pre-determined titer (i.e., the titer at which the same assay conducted without Ab was within the linear range of the assay). Twenty-four µl of pre-diluted psV and 96 µl of pre-diluted Ab were then mixed in the wells of a 96-well polypropylene plate on ice. Additional controls included wells without Ab or psV (‘background’ wells) and wells without Ab that had psV (‘no Ab’ wells). All plates were incubated at RT for 1 hour before transferring to 293TT cells and then all were incubated for an additional 68 hours at 37°C.

At the end of this incubation period, 30 µl of supernatant were removed from each well, transferred to an Immulon 2 HB plate containing 100 µl of AP substrate [4.3 mg/ml Sigma 104 phosphatase substrate (Sigma-Aldrich) in 100 mM sodium bicarbonate buffer, 10 mM magnesium chloride, pH 9.5], and incubated 30 minutes at RT. Absorbance was read at 405 nm. Sample signals were corrected for background and percent neutralization determined, where percent neutralization = [(mean signal_no Ab wells_−signal_Ab wells_)/(mean signal_no Ab wells_)]*100.

### HPV 16 psV ELISA assay

HPV 16 psV were incubated on Immunlon II plates (Thermo Fisher Scientific) overnight at 4°C in PBS. After washing 3× with PBS, plates were blocked for 1 hour with blocker (PBS plus 0.05% Tween-20, 2% non-fat dry milk). Human Abs were diluted in blocking buffer starting at 100 nM or 10 nM, followed by 1∶3 serial dilutions. Plates were incubated at room temperature with gentle shaking for 1 hour followed by washes. Alkaline phosphatase-conjugated goat anti-human IgG, Fc_γ_ specific (Jackson ImmunoResearch Labs, Inc., West Grove, PA; 1∶1000 dilution in blocking buffer), was added to all wells and incubated as before. After washing, plates were developed by the addition of AP substrate and read at 30 minutes at 405 nm. Reagents were added to the plate in 50 µl volumes except for blocker and developer (200 µl).

### Ab sequence analysis

Leader-variable region sequences obtained by primer-specific PCR amplification of heavy and light chain cDNA and subsequent DNA sequencing were submitted to V-QUEST and IgBlast using the default settings, except that for V-QUEST, the number of diversity (D) genes accepted was increased to three. If there was a discrepancy between V gene assignments or number of nucleotide (NT) mutations/amino acid (AA) changes, upon closer inspection V-QUEST typically identified more closely related germline sequences than IgBlast. It should be noted that NT mutations and AA changes in the V gene that overlapped with ‘n’ nucleotides or a given D or joining (J) gene were not counted in the somatic hypermutation statistics.

### Graphs and statistical analyses

Flow cytometry data were analyzed and plotted using FlowJo software (Tree Star, Ashland, Oregon). All other graphs were plotted and prepared using Prism, including any statistical analyses (GraphPad Software, La Jolla, California). IC_50_ neutralization values were determined using Prism's non-linear regression analysis, specifically the dose-response inhibition model and log (inhibitor) vs. response equation. EC_50_ binding values were determined using the sigmoidal (variable slope) equation.

## Supporting Information

Figure S1 **Alexa Fluor 488 (AF488)-conjugation of BPV and HPV 16 psV does not block recognition by type-specific mAbs and generates brightly fluorescent Ag.** (**A**) The anti-HPV 16 murine mAb, H16.V5, and anti-BPV murine mAb, 5B6, equally recognize unconjugated and AF488-conjugated HPV 16 and BPV psV, respectively, in a 293TT neutralization assay. HPV 16 psV are represented by solid pink lines, BPV psV by solid black lines, AF488-HPV 16 by dotted pink lines, and AF488-BPV by dotted black lines. (**B**) To identify optimal staining conditions for flow cytometry, AF488-HPV 16 and AF488-BPV were first titrated on 293TT cells. As shown on the X-axis, optimized amounts of AF488-HPV 16 and AF488-BPV exhibit equal fluorescence intensities, which are baseline resolved from the level of 293TT cell auto-fluorescence.
(PDF)

Figure S2 **Coverage of known leader sequences by our primer sets.** Human leader sequences for the heavy (**A**), kappa (**B**) or lambda (**C**) chains were obtained from the IMGT's LIGM [66] and GENE databases [67]. Specifically, we downloaded functional L-PART1, L-PART1+L-PART2, and L-REGION nucleotide sequences, where L-PART1 is the first of two leader exons in genomic DNA (gDNA) or unspliced coding DNA (cDNA), L-PART2 is the second exon, L-PART1+L-PART2 represents artificially spliced leader sequences, and L-REGION is the leader region coding sequence of spliced cDNA or artificially spliced gDNA. Each line represents a separate leader region allele. Shaded regions indicate leader sequences with homology to our primers.
(PDF)

Figure S3 **Coverage of known heavy chain leader sequences by other primer sets.** As described in the legend for **Figure S2**, each line represents a separate leader region allele for the heavy chain. Shaded regions indicated leader sequences with homology to commonly used primer sets [**37**, **68**, **69**].
(PDF)

Figure S4 **Coverage of known kappa chain leader sequences by other primer sets.** Each line represents a separate leader region allele for the kappa chain. Shaded regions indicated leader sequences with homology to commonly used primer sets [**37]**, [60], [68]–[70].
(PDF)

Figure S5 **Coverage of known lambda chain leader sequences by other primer sets.** Each line represents a separate leader region allele for the lambda chain. Shaded regions indicated leader sequences with homology to commonly used primer sets [**37]**, [60], [68]–[70].
(PDF)

Figure S6 **Purification yields of mAbs from 22 ml of transfected cell supernatant.** All data points represent human mAbs expressed with their native leader, except the point in fuchsia, which represents the yield obtained when an irrelevant mAb was expressed using the AbVec-encoded leader. The observed coefficient of variation between independent transfection and purification experiments was 0.52–8.4%.
(PDF)

Figure S7 **Neutralization potencies of ‘hybrid’ Ab 6 and ‘corrected’ Ab 6.** Hybrid Ab 6 contains C_λ_ allele IGLC1*02 upstream of the XhoI cloning site and C_λ_ allele IGLC2*02 (vector C_λ_ allele) downstream of the cloning site. There are 5 NT differences and 2 AA differences between the IGLC1*02 and IGLC2*02 allele within this short region. We reverted these NT to the vector allele to assess whether they influence expression, folding, or function. We find that these NT do not substantially influence these qualities, for hybrid Ab 6 and corrected Ab 6 (with a full-length IGLC2*02 allele) express similarly and neutralize HPV 16 psV within nearly identical IC_50_ values in the 293TT neutralization assay.
(PDF)

Figure S8 **Neutralization curves of human mAbs at a starting dilution of 50 nM.**
(PDF)

Methods S1 **ELISA to identify purification fractions containing psV.**
(DOCX)

Table S1 **Primers used in this study, including specifications for the amplification PCR (e.g., designation of primer sets, annealing temperatures, etc.).**
(XLSX)
