# Correction: Characteristics of Memory B Cells Elicited by a Highly Efficacious HPV Vaccine in Subjects with No Pre-existing Immunity

**DOI:** 10.1371/journal.ppat.1004612

**Published:** 2014-12-18

**Authors:** The PLOS Pathogens Staff

Table S1 in the published article is missing three primers. Please view the corrected version of Table S1 here for the complete list of primers, with omissions highlighted in yellow.

## Supporting Information

Table S1 **Primers used in this study, including specifications for the amplification PCR (e.g., designation of primer sets, annealing temperatures, etc.).**
(XLSX)
