# Supplementary figures and images for: Characteristics of Memory B Cells Elicited by a Highly Efficacious HPV Vaccine in Subjects with No Pre-existing Immunity
Source: PLoS Pathog. 2014 Oct 16;10(10):e1004461. doi: 10.1371/journal.ppat.1004461 (PMC4199765; doi:10.1371/journal.ppat.1004461)

A

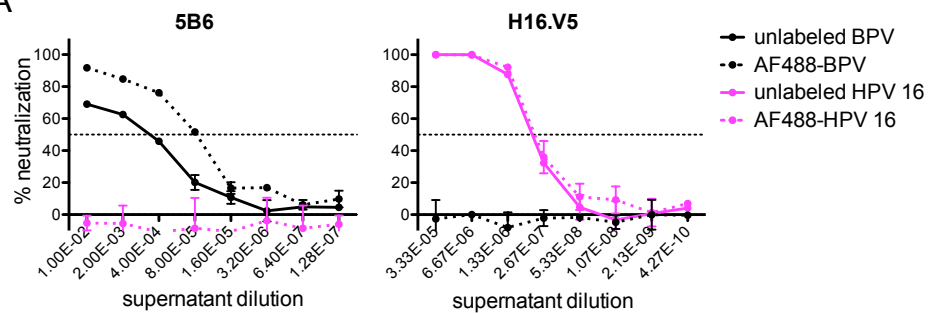

B

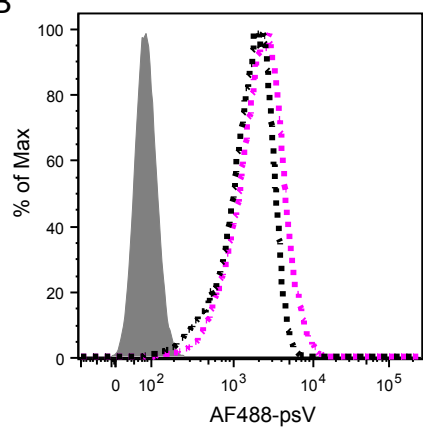

Supplement: Figure S1 — Alexa Fluor 488 (AF488)-conjugation of BPV and HPV 16 psV does not block recognition by type-specific mAbs and generates brightly fluorescent Ag. (A) The anti-HPV 16 murine mAb, H16.V5, and anti-BPV murine mAb, 5B6, equally recognize unconjugated and AF488-conjugated HPV 16 and BPV psV, respectively, in a 293TT neutralization assay. HPV 16 psV are represented by solid pink lines, BPV psV by solid black lines, AF488-HPV 16 by dotted pink lines, and AF488-BPV by dotted black lines. (B) To identify optimal staining conditions for flow cytometry, AF488-HPV 16 and AF488-BPV were first titrated on 293TT cells. As shown on the X-axis, optimized amounts of AF488-HPV 16 and AF488-BPV exhibit equal fluorescence intensities, which are baseline resolved from the level of 293TT cell auto-fluorescence. (PDF) [file ppat.1004461.s001.pdf]

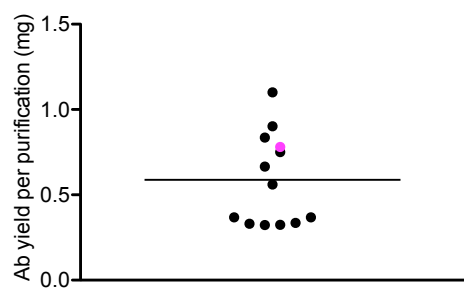

Supplement: Figure S6 — Purification yields of mAbs from 22 ml of transfected cell supernatant. All data points represent human mAbs expressed with their native leader, except the point in fuchsia, which represents the yield obtained when an irrelevant mAb was expressed using the AbVec-encoded leader. The observed coefficient of variation between independent transfection and purification experiments was 0.52–8.4%. (PDF) [file ppat.1004461.s006.pdf]

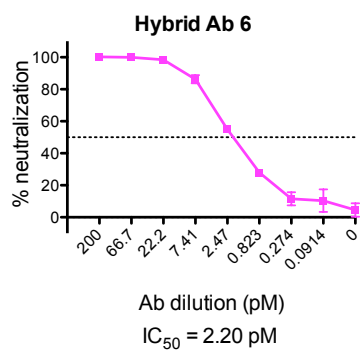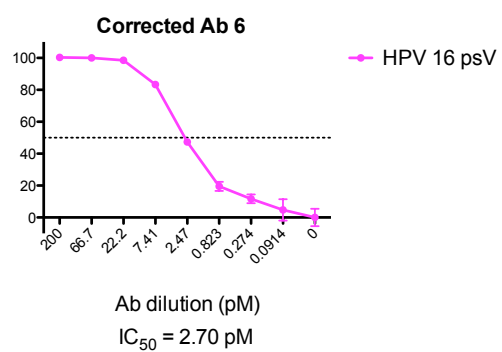

Supplement: Figure S7 — Neutralization potencies of ‘hybrid’ Ab 6 and ‘corrected’ Ab 6. Hybrid Ab 6 contains Cλ allele IGLC1*02 upstream of the XhoI cloning site and Cλ allele IGLC2*02 (vector Cλ allele) downstream of the cloning site. There are 5 NT differences and 2 AA differences between the IGLC1*02 and IGLC2*02 allele within this short region. We reverted these NT to the vector allele to assess whether they influence expression, folding, or function. We find that these NT do not substantially influence these qualities, for hybrid Ab 6 and corrected Ab 6 (with a full-length IGLC2*02 allele) express similarly and neutralize HPV 16 psV within nearly identical IC50 values in the 293TT neutralization assay. (PDF) [file ppat.1004461.s007.pdf]

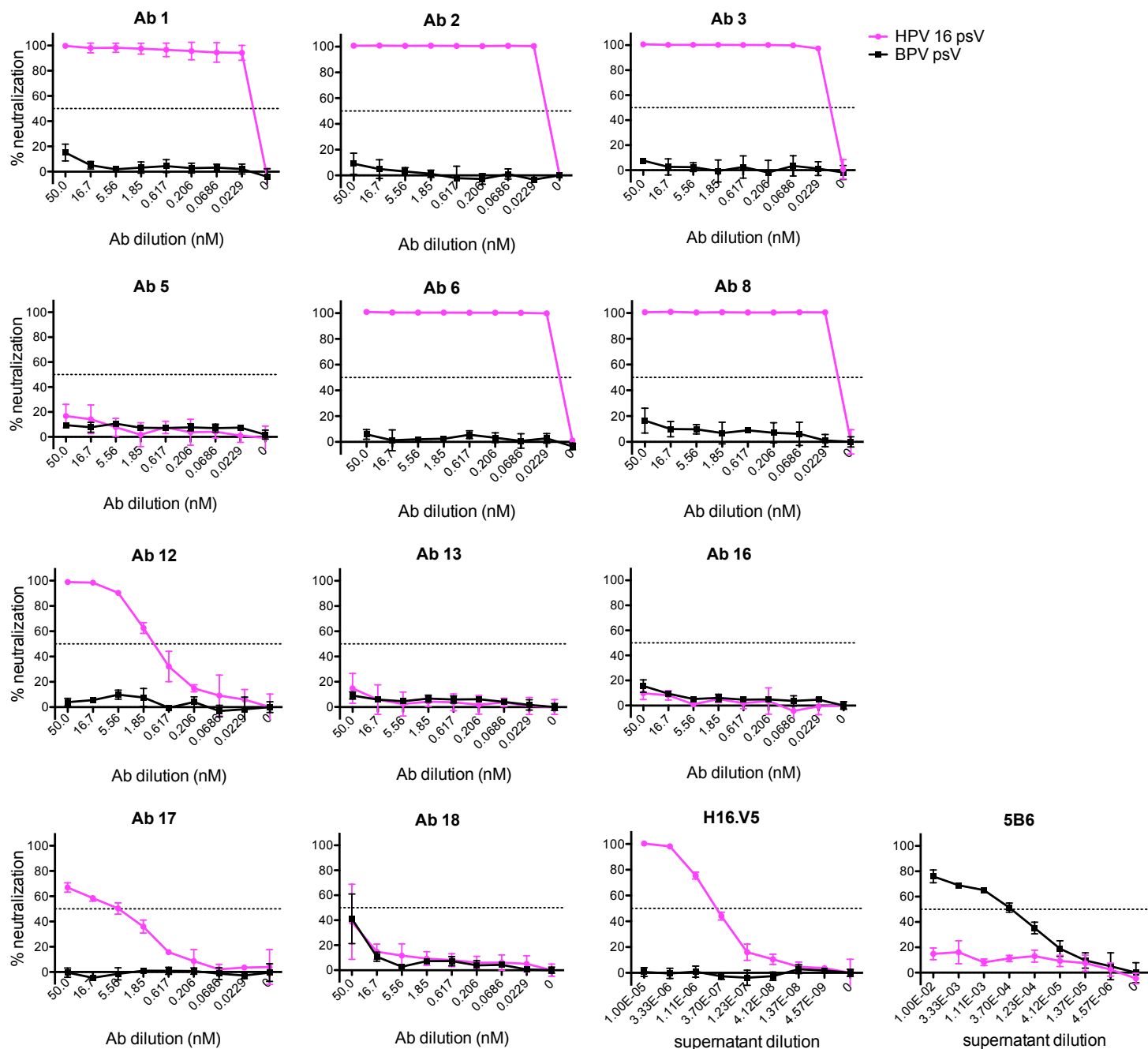

Supplement: Figure S8 — Neutralization curves of human mAbs at a starting dilution of 50 nM. (PDF) [file ppat.1004461.s008.pdf]
