# Supplementary material for: Characteristics of Memory B Cells Elicited by a Highly Efficacious HPV Vaccine in Subjects with No Pre-existing Immunity
Source: PLoS Pathog. 2014 Oct 16;10(10):e1004461. doi: 10.1371/journal.ppat.1004461 (PMC4199765; doi:10.1371/journal.ppat.1004461)
Supplement: Figure S2 — Coverage of known leader sequences by our primer sets. Human leader sequences for the heavy (A), kappa (B) or lambda (C) chains were obtained from the IMGT's LIGM [66] and GENE databases [67]. Specifically, we downloaded functional L-PART1, L-PART1+L-PART2, and L-REGION nucleotide sequences, where L-PART1 is the first of two leader exons in genomic DNA (gDNA) or unspliced coding DNA (cDNA), L-PART2 is the second exon, L-PART1+L-PART2 represents artificially spliced leader sequences, and L-REGION is the leader region coding sequence of spliced cDNA or artificially spliced gDNA. Each line represents a separate leader region allele. Shaded regions indicate leader sequences with homology to our primers. (PDF) [file ppat.1004461.s002.pdf]

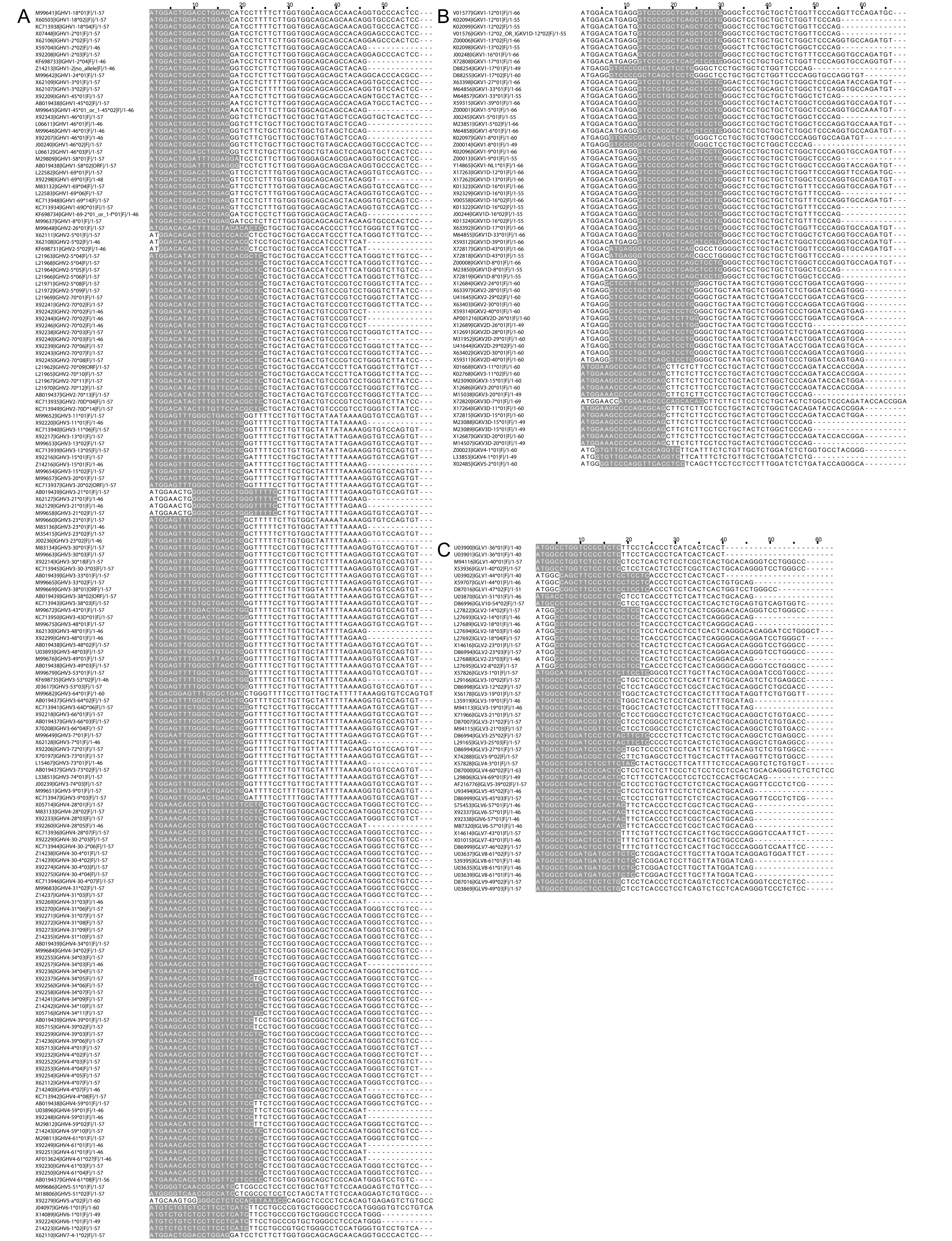
A
B
C
