# Supplementary material for: Characteristics of Memory B Cells Elicited by a Highly Efficacious HPV Vaccine in Subjects with No Pre-existing Immunity
Source: PLoS Pathog. 2014 Oct 16;10(10):e1004461. doi: 10.1371/journal.ppat.1004461 (PMC4199765; doi:10.1371/journal.ppat.1004461)
Supplement: Methods S1 — ELISA to identify purification fractions containing psV. (DOCX) [file ppat.1004461.s009.docx]

**Supporting Information**

**Methods S1. ELISA to identify purification fractions containing psV.**

Three μl of each fraction were diluted into 97 μl PBS in a 96-well Immulon™ 2 HB plate (Thermo Fisher Scientific). Plates were incubated, covered, while shaking, for 1-2 hours at room temperature (RT) or overnight at 4°C. Plates were washed three times with PBS-0.05% Tween-20 and blocked with 4% (w/v) non-fat dry milk (NFDM)/PBS-0.05% Tween-20 for 1-2 hours at RT, while covered, with shaking. Blocking buffer was discarded without washing, and either H16.V5 or 5B6 diluted 1:1000 in 100 μl 0.4% (w/v) NFDM/PBS-0.05% Tween-20 were incubated with each AF488-conjugated HPV 16 psV (AF488-HPV 16) purification fraction or each AF488-conjugated BPV psV (AF488-BPV) purification fraction, respectively, for 1 hour at RT, while covered, with shaking. Plates were then washed three times and incubated with AP-conjugated anti-mouse IgG Fc specific (Jackson ImmunoResearch Laboratories, West Grove, Pennsylvania) at a dilution of 1:5000 in 100 μl 0.4% NFDM/PBS-0.05% Tween-20 for 0.5 – 1 hour at RT, while covered, with shaking. A final three washes were performed and then 100 μl of AP substrate [4.3 mg/ml Sigma 104 phosphatase substrate in 100 mM sodium bicarbonate buffer, 10 mM magnesium chloride, pH 9.5] were added to each well, and plates read until an absorbance of 1.0 at 405 nm was achieved.
