# Supplementary material for: Characteristics of Memory B Cells Elicited by a Highly Efficacious HPV Vaccine in Subjects with No Pre-existing Immunity
Source: PLoS Pathog. 2014 Oct 16;10(10):e1004461. doi: 10.1371/journal.ppat.1004461 (PMC4199765; doi:10.1371/journal.ppat.1004461)

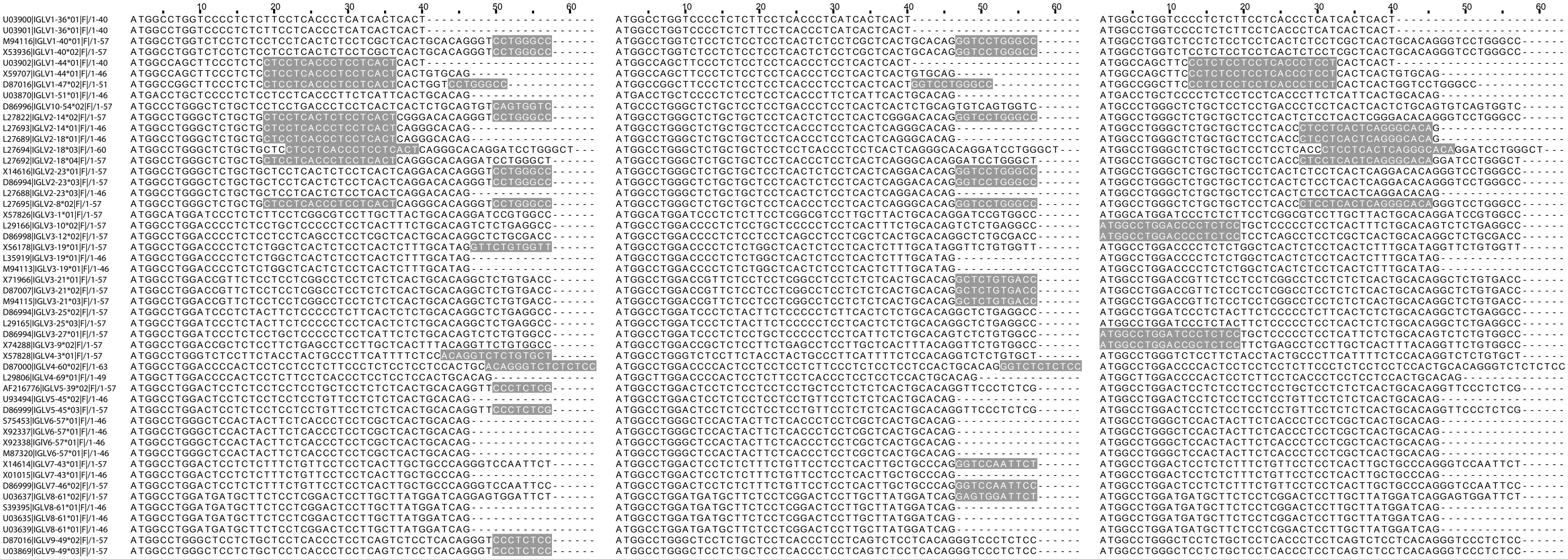

Supplement: Figure S5 — Coverage of known lambda chain leader sequences by other primer sets. Each line represents a separate leader region allele for the lambda chain. Shaded regions indicated leader sequences with homology to commonly used primer sets [37] , [60], [68]–[70]. (PDF) [file ppat.1004461.s005.pdf]
